# Supplementary material for: MetaRibo-Seq measures translation in microbiomes
Source: Nat Commun. 2020 Jun 29;11:3268. doi: 10.1038/s41467-020-17081-z (PMC7324362; doi:10.1038/s41467-020-17081-z)
Supplement: Supplementary file 10 — Supplementary Data 7 [file 41467_2020_17081_MOESM10_ESM.zip › File2/Confidence_High_Taxonomy/386459_out.krona.html]

Javascript must be enabled to view this page.

members
magnitude
magnitudeUnassigned
count
unassigned
taxon
rank

386459\_out

216

216
2
superkingdom

976
phylum
216

200643
class
216

216
171549
order

215
815
family

816
genus
215

46506
species
1

SRS971276\_contig\_number\_975

47678
species

SRS011134\_contig\_number\_3526SRS011302\_contig\_number\_12855SRS012273\_contig\_number\_41426SRS012849\_contig\_number\_21283SRS012969\_contig\_number\_6734SRS013098\_contig\_number\_5353SRS013476\_contig\_number\_23163SRS013521\_contig\_number\_6141SRS013687\_contig\_number\_12981SRS013951\_contig\_number\_28949SRS013965\_contig\_number\_37046SRS014235\_contig\_number\_49062SRS014287\_contig\_number\_10698SRS014313\_contig\_number\_4236SRS014412\_contig\_number\_12473SRS014459\_contig\_number\_37100SRS014613\_contig\_number\_4474SRS014948\_contig\_number\_15873SRS015065\_contig\_number\_24802SRS015264\_contig\_number\_10821SRS015663\_contig\_number\_10033SRS015694\_contig\_number\_18639SRS015782\_contig\_number\_34434SRS015960\_contig\_number\_308SRS016018\_contig\_number\_5426SRS016203\_contig\_number\_5884SRS016335\_contig\_number\_12220SRS016541\_contig\_number\_1798SRS017103\_contig\_number\_5091SRS017191\_contig\_number\_3278SRS017433\_contig\_number\_7573SRS017701\_contig\_number\_10296SRS017821\_contig\_number\_20442SRS018049\_contig\_number\_230SRS018133\_contig\_number\_11008SRS018313\_contig\_number\_8293SRS018427\_contig\_number\_2372SRS018541\_contig\_number\_10264SRS018623\_contig\_number\_12604SRS018836\_contig\_number\_21672SRS018888\_contig\_number\_1349SRS018936\_contig\_number\_9696SRS019161\_contig\_number\_1447SRS019178\_contig\_number\_2180SRS019267\_contig\_number\_6670SRS019381\_contig\_number\_4662SRS019397\_contig\_number\_339SRS019601\_contig\_number\_2195SRS019685\_contig\_number\_7801SRS019910\_contig\_number\_10817SRS020233\_contig\_number\_12715SRS020271\_contig\_number\_669SRS020328\_contig\_number\_31499SRS020394\_contig\_number\_13393SRS021219\_contig\_number\_3013SRS022071\_contig\_number\_20758SRS022137\_contig\_number\_12863SRS023526\_contig\_number\_438SRS023583\_contig\_number\_591SRS023715\_contig\_number\_20112SRS023914\_contig\_number\_1372SRS024132\_contig\_number\_20473SRS024331\_contig\_number\_12391SRS024492\_contig\_number\_11410SRS024625\_contig\_number\_908SRS042966\_contig\_number\_20202SRS043001\_contig\_number\_7039SRS043667\_contig\_number\_1496SRS043701\_contig\_number\_3609SRS043768\_contig\_number\_17959SRS043841\_contig\_number\_3298SRS044535\_contig\_number\_6150SRS045004\_contig\_number\_9581SRS045244\_contig\_number\_3434SRS045645\_contig\_number\_13888SRS045826\_contig\_number\_17130SRS046068\_contig\_number\_701SRS047014\_contig\_number\_27311SRS047044\_contig\_number\_12800SRS047433\_contig\_number\_13187SRS048060\_contig\_number\_10157SRS048164\_contig\_number\_31573SRS049712\_contig\_number\_20562SRS049773\_contig\_number\_1816SRS049995\_contig\_number\_16945SRS050026\_contig\_number\_8711SRS050299\_contig\_number\_6137SRS050422\_contig\_number\_26341SRS050752\_contig\_number\_24653SRS050801\_contig\_number\_1527SRS050941\_contig\_number\_14992SRS050998\_contig\_number\_11861SRS051031\_contig\_number\_1454SRS051882\_contig\_number\_1486SRS052078\_contig\_number\_389SRS052697\_contig\_number\_24069SRS053398\_contig\_number\_10818SRS053573\_contig\_number\_553SRS053649\_contig\_number\_1440SRS054352\_contig\_number\_15490SRS054905\_contig\_number\_2336SRS056259\_contig\_number\_5941SRS056273\_contig\_number\_11004SRS058723\_contig\_number\_6241SRS058770\_contig\_number\_27124SRS062427\_contig\_number\_4085SRS062654\_contig\_number\_12829SRS063370\_contig\_number\_1910SRS063518\_contig\_number\_38896SRS064276\_contig\_number\_25679SRS064557\_contig\_number\_75SRS064757\_contig\_number\_contig-100\_556.133774SRS064973\_contig\_number\_1501SRS065176\_contig\_number\_19SRS065397\_contig\_number\_10305SRS065504\_contig\_number\_11468SRS074964\_contig\_number\_contig-100\_572.58216SRS075078\_contig\_number\_8636SRS075773\_contig\_number\_28102SRS075821\_contig\_number\_21174SRS075878\_contig\_number\_12371SRS075984\_contig\_number\_4391SRS077024\_contig\_number\_6367SRS077086\_contig\_number\_5075SRS077294\_contig\_number\_16283SRS077335\_contig\_number\_1496SRS077392\_contig\_number\_20841SRS077454\_contig\_number\_2148SRS077502\_contig\_number\_4514SRS077589\_contig\_number\_7180SRS077849\_contig\_number\_23124SRS078176\_contig\_number\_17670SRS078242\_contig\_number\_12990SRS078419\_contig\_number\_7113SRS078665\_contig\_number\_3618SRS098571\_contig\_number\_13521SRS098644\_contig\_number\_1509SRS101268\_contig\_number\_755SRS101376\_contig\_number\_19775SRS103987\_contig\_number\_1173SRS1041031\_contig\_number\_22079SRS1041032\_contig\_number\_29SRS1041039\_contig\_number\_21222SRS1041090\_contig\_number\_5653SRS1041091\_contig\_number\_9703SRS1041092\_contig\_number\_13446SRS1041118\_contig\_number\_1942SRS1041134\_contig\_number\_2771SRS1041137\_contig\_number\_15791SRS1041138\_contig\_number\_7233SRS1041139\_contig\_number\_4377SRS1041141\_contig\_number\_7013SRS1041145\_contig\_number\_8105SRS1041157\_contig\_number\_1159SRS104311\_contig\_number\_12138SRS104327\_contig\_number\_4300SRS104400\_contig\_number\_35385SRS104975\_contig\_number\_contig-100\_442.130865SRS105082\_contig\_number\_contig-100\_284.134989SRS105153\_contig\_number\_12539SRS1055022\_contig\_number\_4999SRS1055049\_contig\_number\_15068SRS1055076\_contig\_number\_1132SRS142542\_contig\_number\_7507SRS142599\_contig\_number\_13662SRS142781\_contig\_number\_1141SRS142890\_contig\_number\_19286SRS142923\_contig\_number\_14330SRS143181\_contig\_number\_14156SRS143342\_contig\_number\_12557SRS143523\_contig\_number\_19897SRS143598\_contig\_number\_3464SRS143722\_contig\_number\_10556SRS143876\_contig\_number\_38506SRS143895\_contig\_number\_9853SRS144183\_contig\_number\_contig-100\_482.170415SRS144362\_contig\_number\_2165SRS144506\_contig\_number\_23656SRS144603\_contig\_number\_15487SRS145308\_contig\_number\_536SRS145497\_contig\_number\_215SRS147039\_contig\_number\_4339SRS147346\_contig\_number\_2157SRS147425\_contig\_number\_1331SRS147557\_contig\_number\_20256SRS147614\_contig\_number\_10454SRS147977\_contig\_number\_598SRS148091\_contig\_number\_7925SRS148196\_contig\_number\_34694SRS148253\_contig\_number\_3903SRS148319\_contig\_number\_4735SRS148424\_contig\_number\_26481SRS148721\_contig\_number\_55544SRS148817\_contig\_number\_2746SRS149244\_contig\_number\_1812SRS149879\_contig\_number\_21211SRS150029\_contig\_number\_4618SRS892882\_contig\_number\_16157SRS893230\_contig\_number\_8389SRS893252\_contig\_number\_7705SRS893279\_contig\_number\_contig-100\_538.98565SRS893285\_contig\_number\_271SRS893292\_contig\_number\_3120SRS893295\_contig\_number\_1807SRS893300\_contig\_number\_8406SRS893327\_contig\_number\_1027SRS893366\_contig\_number\_18360SRS893378\_contig\_number\_9830SRS893382\_contig\_number\_2673SRS893383\_contig\_number\_10262
210


SRS011084\_contig\_number\_15550SRS021484\_contig\_number\_953
2
1263037
species

1

SRS146813\_contig\_number\_19013
species
820

1

SRS098827\_contig\_number\_8670
species
1262750

1
family
1853231

genus
283168
1

1

SRS149784\_contig\_number\_27024
626933
species
